# Impaired Quality of Life and Need for Palliative Care in a German Cohort of Advanced Parkinson’s Disease Patients

**DOI:** 10.3389/fneur.2018.00120

**Published:** 2018-03-06

**Authors:** Martin Klietz, Amelie Tulke, Lars H. Müschen, Lejla Paracka, Christoph Schrader, Dirk W. Dressler, Florian Wegner

**Affiliations:** ^1^Department of Neurology, Hannover Medical School, Hannover, Germany

**Keywords:** advanced Parkinson’s disease, palliative care, end-of-life care, quality of life, non-motor symptoms

## Abstract

**Background:**

Parkinson’s disease (PD) is the second most frequent neurodegenerative disease of the elderly. Patients suffer from various motor and non-motor symptoms leading to reduced health-related quality of life (HRQOL) and an increased mortality. Their loss of autonomy due to dementia, psychosis, depression, motor impairments, falls, and swallowing deficits defines a phase when palliative care interventions might help to sustain or even improve quality of life.

**Objective:**

The aim of this study was to investigate the current status of palliative care implementation and quality of life in a local cohort of advanced PD patients in order to frame and improve future care.

**Methods:**

76 geriatric patients with advanced idiopathic PD meeting the inclusion criteria for palliative care interventions were clinically evaluated by neurological examination using Movement Disorders Society Unified Parkinson’s Disease Rating Scale, Barthel Index, Montreal Cognitive Assessment Test, and a structured interview concerning palliative care implementation.

**Results:**

HRQOL is severely reduced in our cohort of geriatric advanced PD patients. We found motor deficits, impairment of activities of daily living, depression, and cognitive decline as most relevant factors determining decreased HRQOL. Only 2.6% of our patients reported present implementation of palliative care. By contrast, 72% of the patients indicated an unmet need for palliative care.

**Conclusion:**

Quality of life is dramatically affected in advanced PD patients. However, we found palliative care to be implemented extremely rare in their treatment concept. Therefore, geriatric patients suffering from advanced PD should be enrolled for palliative care to provide adequate and holistic treatment which may improve or sustain their quality of life.

## Introduction

Parkinson’s disease (PD) is the second most frequent neurodegenerative disease of the elderly (1). Despite good treatment options in early disease with fair sustainment of quality of life, in advanced stages of PD therapy can be challenging and quality of life is dramatically reduced (2–4). Additional to motor symptoms such as rigidity, tremor, bradykinesia, and postural instability, patients are affected by non-motor symptoms such as depression, obstipation, urinary incontinence, psychiatric disease, and cognitive deficits (5). This symptom burden markedly affects quality of life measured as health-related quality of life (HRQOL) of PD patients and induces caregiver burden as well (6–11). HRQOL and influencing factors are well characterized in early PD, especially dementia is related to poor quality of life and shortened survival (12). Comparatively little is known about determining factors of HRQOL in patients with advanced PD suitable for palliative care interventions (13). Advanced PD patients live with a high symptom burden and an increased risk of mortality, thus, meeting criteria for palliative care. Caregivers of these patients have a high caregiver burden showing a high incidence of depressive symptoms in a small cohort in Germany (caregivers of 20 advanced PD patients) (10).

For advanced PD patients, early implementation of palliative care (e.g., hospice and nursing service, advanced care planning, like feeding in the case of swallowing deficits, airway management, and symptom focused therapy) and end-of-life planning may be the key to adequate treatment and sustainment of quality of life (14). Still, palliative care is offered rarely to these patients (15). Geriatric PD patients are seldomly admitted to a hospice and often die in a hospital because adequate palliative care settings are not provided at home (16). In geriatric patients with advanced PD, drug therapy is often limited by side effects and contraindications due comorbidity. Thus, sufficient symptomatic therapy provided by palliative care concepts can be crucial.

In contrast to the emerging acknowledgment and integration of palliative care in other medical fields, the implementation of palliative care remains uncommon in the treatment of advanced PD in Germany (17). Currently, there are no data available on palliative care implementation in advanced PD treatment and its effects on HRQOL in Germany. Miyasaki et al. report good relief of symptoms in a Canadian study using the Edmonton Symptom Assessment System for PD for palliative care interventions in advanced PD patients (14). Another study reports clinical palliative care interventions for patients with atypical parkinsonisms (18).

To specify the needs of geriatric PD patients in advanced stages of disease, we performed a clinical study investigating HRQOL, its influencing factors, and the extent of palliative care implementation in a local cohort. The aim of this study was to investigate the current status of palliative care implementation in the German health system and to evaluate HRQOL and its influencing factors in this particular group of patients, in order to frame and improve future interventions.

## Patients and Methods

We obtained approval from the local Ethics Committee of Hannover Medical School (No. 3123-2016), and patients or their caregivers gave written informed consent. 76 patients with idiopathic PD were recruited from (1) our movement disorder outpatient clinic, (2) our neurological wards, (3) local PD patient support groups, and (4) outpatient neurologists in the region of Hannover, Germany. PD patients who had been admitted *via* the emergency department to our neurological wards were not included in our study until successful treatment had led to a stable condition again. Inclusion criteria for geriatric advanced PD were defined as Hoehn and Yahr stage (H&Y) 3 or more (scored during the on period), 65 years of age or older, disease duration of at least 5 years, and loss of autonomy due to PD (19) because these patients are most likely suitable for palliative care interventions. Patients with atypical parkinsonism and those suffering from much more troublesome comorbidities were excluded from this study.

Participants were examined using MDS-UPDRS (assessment of PD symptoms in the clinical on), MoCA test [cognitive screening test, range from 0 to 30 points, 30–26 points were considered as normal cognitive function, 25–21 points as mild cognitive impairment, and below 21 points as suspicious for dementia (20, 21)], Barthel Index (general performance, activities of daily living), and PDQ-39 (HRQOL specifically constructed and validated for PD). To avoid anosognosia affecting HRQOL measurement, we included demented PD patients only after involvement of the corresponding caregivers who were able to exclude anosognosia as relevant confounder. Furthermore, a structured interview was performed to define the need for and evaluate the frequency of palliative care in these patients. In detail, we evaluated patients’ current accommodation and care, such as living at their own home either with or without help by professional caregivers or residing in a nursing home or in a hospice. Participants were asked if they had an advance directive including specific restrictions of therapy and whether they had determined a health-care proxy or someone granted general power of attorney. Patients were interviewed on current implementation of palliative care, such as an outpatient palliative care or hospice service. They also stated if palliative care matters had been addressed by their physicians in the past and whether they had discussed palliative care matters within their family. Finally, they were asked if and with whom—their doctors, family, and friends or others—they wished to discuss palliative care matters and where they would wish to die. Deep brain stimulation, duodopa treatment, and subcutaneous apomorphine therapy as well as oral PD medication was noted for each patient and the equivalence dosage of levodopa was calculated according to Tomlinson et al. (22). A full medical history of all participants was taken, if available, comorbidities and medication were noted from the most updated physician’s letter.

Statistical analysis was performed using Graphpad Prism 5.00 (San Diego, CA, USA). Data were analyzed by calculating mean, SD, and range. Comparison between two groups was performed by unpaired Student’s *t*-test. Comparison between more than two groups was performed by one-way ANOVA and Newman–Keuls post-test. Correlations were calculated by linear regression analysis (*r*^2^ = 1 − SS_reg_/SS_tot_, where SS_reg_ is the variance (sum of squares) of the data of the linear regression model and SS_tot_ is the total variance of the *Y* values) and using the sample Pearson correlation coefficient. A *p*-value lower than 0.05 was considered as significant.

## Results

### Quality of Life in Patients with Advanced Parkinson’s Disease

In our study, we included 76 patients with advanced PD, 53.9% were female. Our patients presented with a mean H&Y of 4.0 (SD 0.7; range 3–5), and the mean age was 76 years (SD 6.1; range 65–89 years). Mean disease duration was 17.3 years (SD 7.3; range 5–38 years). Participants suffered from severe restrictions in the activities of daily living measured by the Barthel Index (mean 61.8 points; SD 25.4; range 10–100) and by the MDS-UPDRS part II (mean 31.4; SD 8.1; range 16–48).

68 out of 76 PD patients (89.5%) presented with cognitive deficits estimated by a MoCA test score below 26 points and a mean score of 18.4 points (SD 7.8; range 0–30). 50.0% of patients scored below 21 points in the MoCA test which is considered to be highly suggestive for dementia (20, 21). Before study participation, some of our patients (22.4%) had already been diagnosed with dementia according to the S3 guideline for dementia of the German Society of Neurology based on (23). To our surprise, antidementive drugs had been prescribed to only two patients (2.6%). Psychiatric symptoms, measured by the MDS-UPDRS part I item 1.1 “hallucinations and psychosis” (score equal to 2 or more), were reported by 34 patients (44.7%). Almost the same proportion of patients (38.2%) was prescribed neuroleptic drugs (e.g., clozapine or quetiapine).

Depressive mood was present in 52.6% of our patients (40/76) measured by the MDS-UPDRS part I item 1.3 “depressed mood” (score equal to 2 or more). However, a diagnosis of depression in the patients’ previous medical documents had been established in only 10.5%, whereas antidepressive medication was prescribed to 15.8% of patients. More extensive and time consuming additional assessments of specific depression and anxiety symptoms could not be performed in this study due to the limited general condition of the patients.

Our patients presented with severe motor impairment determined by the MDS-UPDRS III (60.8 points; SD 16; range 24–96) in the clinical examination. In the MDS-UPDRS part IV, 64.5% of patients reported dyskinesias and 48.7% complained about functional impairments due to dyskinesias. Off-phases were present in 75% of the patients with functional impairment of daily activities. Dystonia in the off-phase was reported by 26.3% of the patients.

The PDQ-39 scale HRQOL was drastically reduced (mean 50.8%; SD 12.4%; range 16.7–75%). Patient characteristics are summarized in Table 1.

**Table 1:** Patient characteristics (*n* = 76).

	Mean	SD	Min	Max
Age	75.5	6.1	65	89
Sex			Male 46.1%	Female 53.9%
Barthel Index	61.8	25.4	10	100
H&Y	4.0	0.7	3	5
Disease duration in years	17.3	7.3	5	38
MDS-UPDRS I	20.5	6.3	9	37
MDS-UPDRS I “cognitive impairment”	1.5	1.3	0	4
MDS-UPDRS I “depressed mood”	1.8	1.0	0	4
MDS-UPDRS I “hallucinations and psychosis”	1.3	1.4	0	4
MDS-UPDRS II	31.4	8.1	16	48
MDS-UPDRS III	60.8	16	24	96
MDS-UPDRS IV	8.5	5.1	0	17
MDS-UPDRS IV dyskinesia duration	1.1 (25–50% of the day)	1.1	0	4
MDS-UPDRS IV dyskinesia functional impairment	1.1	1.4	0	4
MDS-UPDRS IV off-phase duration	1.2 (25–50% of the day)	1.9	0	4
MDS-UPDRS IV functional impairment of off-phases	2.3	1.6	0	4
MDS-UPDRS IV off-dystonia	0.6	1.2	0	4
MoCA	18.4	7.8	0	30
PDQ-39 (%)	50.8	12.4	16.7	75
LED (mg)	1,103	541	275	2,552

*H&Y, Hoehn and Yahr stage; MDS-UPDRS, Movement Disorders Society Unified Parkinson’s Disease Rating Scale part I-IV; MoCA, Montreal Cognitive Assessment test; PDQ-39, Parkinson’s Disease Quality of life form 39; LED, calculated l-DOPA equivalence dosage*.

**Figure 1:**
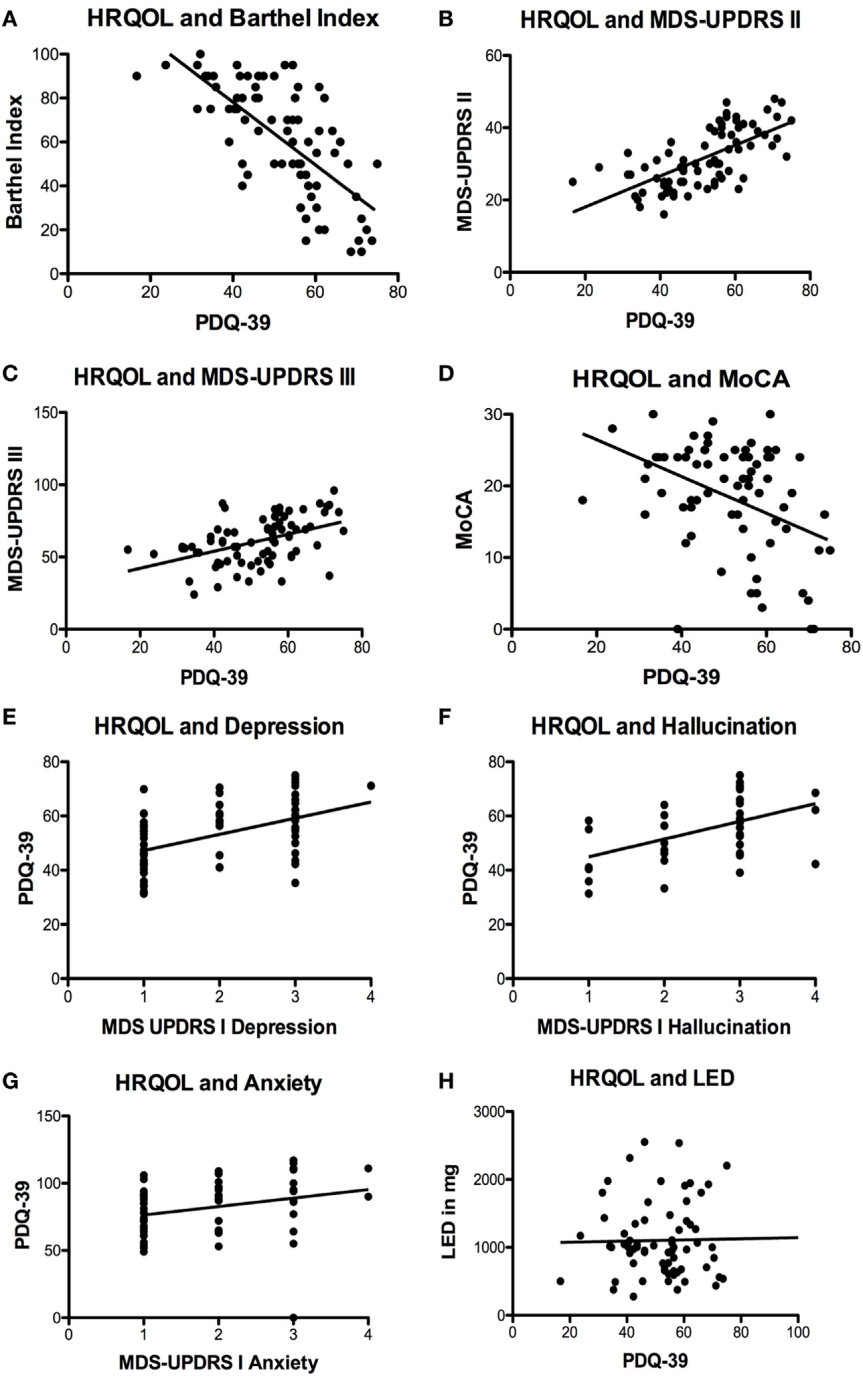
Significant correlations of health-related quality of life (HRQOL) with different scales und symptoms. **(A)** Negative correlation of HRQOL and Barthel Index (*p* < 0.0001; *r* = −0.6946; *r*^2^ = 0.4825). **(B)** Correlation of HRQOL with Movement Disorders Unified Parkinson’s Disease Rating Scale (MDS-UPDRS) part II (*p* < 0.0001; *r* = 0.6586; *r*^2^ = 0.4338) and **(C)** MDS-UPDRS part III (*p* < 0.0001; *r* = 0.4562; *r*^2^ = 0.2081). **(D)** Negative correlation of HRQOL and Montreal Cognitive Assessment test (MoCA) (*p* = 0.0002; *r* = −0.4136; *r*^2^ = 0.1711). **(E)** Correlations of HRQOL with MDS-UPDRS part I item “depressed mood” (*p* < 0.0001; *r* = 0.4862; *r*^2^ = 0.2364) and **(F)** MDS-UPDRS part I item hallucinations and psychosis (*p* = 0.0018; *r* = 0.4841; *r*^2^ = 0.2344) and **(G)** MDS-UPDRS item “anxious mood” (*p* = 0.0431; *r* = 0.2689; *r*^2^ = 0.0723). **(H)** Non-significant correlation of HRQOL and levodopa equivalence dosage (LED) (*p* = 0.5035; *r* = 0.0825; *r*^2^ = 0.0068).

We found highly significant correlations between HRQOL and the activities of daily living measured by the Barthel Index (*p* < 0.0001; *r* = −0.6946; *r*^2^ = 0.4825; Figure 1A), the MDS-UPDRS part II (*p* < 0.0001; *r* = 0.6586; *r*^2^ = 0.4338; Figure 1B), and motor impairment evaluated by MDS-UPDRS part III (*p* < 0.0001; *r* = 0.4562; *r*^2^ = 0.2081; Figure 1C). MDS-UPDRS part I did not correlate in total score with HRQOL (*p* = 0.12; *r* = 0.2049; *r*^2^ = 0.0420); however, the items “depressed mood” (*p* < 0.0001; *r* = 0.4862; *r*^2^ = 0.2364; Figure 1E), “hallucinations and psychosis” (*p* = 0.0018; *r* = 0.4841; *r*^2^ = 0.2344; Figure 1F), and “anxious mood” (*p* = 0.0431; *r* = 0.2689; *r*^2^ = 0.0723; Figure 1G) correlated significantly with the PDQ-39. HRQOL correlated significantly with cognitive deficits measured by MoCA test (*p* = 0.0002; *r* = −0.4136; *r*^2^ = 0.1711; Figure 1D) and the item “cognitive impairment” of MDS-UPDRS part I (*p* < 0.0001; *r* = −0.5833; *r*^2^ = 0.3402).

We also correlated the Barthel Index and MoCA scale with the MDS-UPDRS scores to check for co-correlations. Scores from the Barthel Index correlated significantly with MDS-UPDRS part I (*p* < 0.0001; *r* = 0.5177; *r*^2^ = 0.2680), part II (*p* < 0.0001; *r* = 0.7601; *r* = 0.5778) and part III (*p* < 0.0001; *r* = 0.6920; *r*^2^ = 0.4789), interestingly, they did not correlate with motor complications in the MDS-UPDRS part IV (*p* = 0.9910; *r* = 0.0013; *r*^2^ < 0.0001). Concerning the MoCA score of the patients we found significant correlations with the MDS UPDRS part I (*p* < 0.0001; *r* = −0.4843; *r*^2^ = 0.2345), part II (*p* < 0.0001; *r* = −0.5160; *r*^2^ = 0.2663) and III (*p* < 0.0001; *r* = −0.7163; *r*^2^ = 0.5131) but not with part IV (*p* = 0.2849; *r* = −0.1241; *r*^2^ = 0.0154). Additionally, we found a significant correlation of MoCA scores and the MDS-UPDRS part I items “anxious mood” (*p* = 0.0063; *r* = −0.3108; *r*^2^ = 0.0966) and “hallucinations and psychosis” (*p* = 0.0011; *r* = −0.3667; *r*^2^ = 0.1345).

Regarding motor complications measured by the MDS-UPDRS part IV and their impact on HRQOL, we found no correlation of dyskinesias and HRQOL (MDS-UPDRS IV item 1, *p* = 0.9342; *r* = 0.0096; *r*^2^ < 0.0001 and 2, *p* = 0.3794; *r* = 0.1029; *r*^2^ = 0.0106). The frequency of off-time correlated significantly with HRQOL (*p* = 0.0339; *r* = 0.2454; *r*^2^ = 0.0602); however, the functional impairment of off-time (item 4, *p* = 0.1267; *r* = 0.1779; *r*^2^ = 0.0316) and the complexity of off-phases (MDS-UPDRS IV item 5, *p* = 0.1480; *r* = 0.1686; *r*^2^ = 0.0284) did not correlate with HRQOL. Off-dystonia correlated significantly with HRQOL (*p* = 0.0490; *r* = 0.2280; *r*^2^ = 0.0520).

Treatment regimens markedly differed between individual patients. The number of prescribed PD drugs ranged from one to six groups of medication [levodopa + decarboxylase inhibitor; dopamine agonist; MAO_B_ inhibitor (14.5%); safinamide (8.7%); amantadine (17.6%); COMT inhibitor (40.1%)]. The vast majority received levodopa therapy (98.5%). More than one-third was treated with additional dopamine agonists (41.2%). Interestingly, the calculated equivalence dosage of levodopa did not correlate with HRQOL at all (*p* = 0.5035; *r* = 0.0825; *r*^2^ = 0.0068; Figure 1H). We included 14 patients with DBS (18.4%), 9 patients with duodopa intrajejunal therapy (11.8%) and 3 patients with apomorphine subcutaneous pump (3.9%). No significant differences in HRQOL and motor impairment were found in patients with DBS or duodopa therapy compared to each other and to the cohort with oral medication only (*p* > 0.05, one-way ANOVA and Newman–Keuls post-test). Due to the low number of patients, we did not calculate any data for the apomorphine group.

In the medical documents of our PD patients, we screened for systemic diseases. Note, we excluded patients predominantly suffering from another severe disease than PD. We found 30.3% of patients to be diagnosed with arterial hypertension. Cardiovascular disease, excluding arterial hypertension, was present in 27.6% of the cohort. Diabetes type 1 or 2 was diagnosed in 7.9%; other endocrine diseases, such as hypothyreosis, were present in 13.2% of the patients.

### Palliative Care in Advanced Parkinson’s Disease

Approximately half of our patients managed to schedule regular appointments with an outpatient neurologist. Most neurological consultations of the other patients had to be done *via* the emergency department.

**Figure 2:**
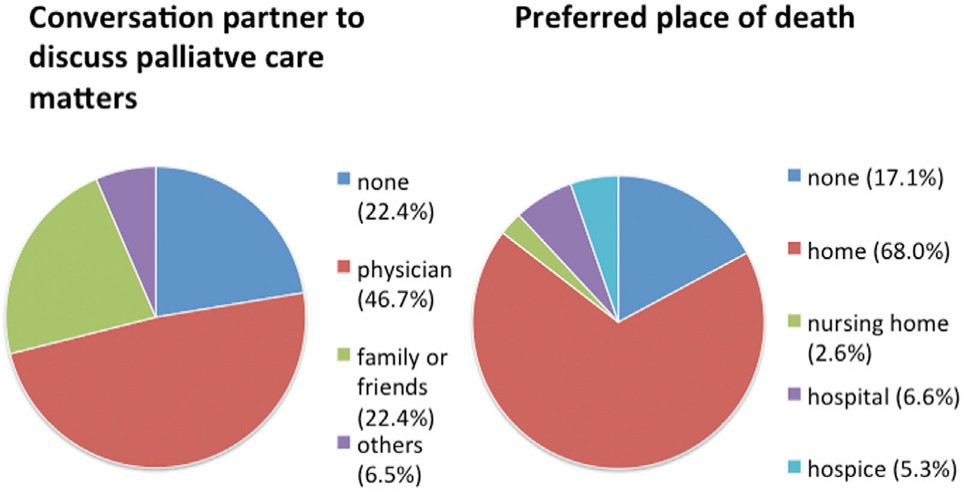
Patients’ wishes in regard to palliative care concerning communication partner and place of death. *N* = 76 Parkinson’s disease patients.

We found 70% of our patients to have an advance directive and a health-care proxy. However, hardly any patient was provided with additional palliative care at their home or had ever consulted their treating physicians on palliative care matters (Table 2). Overall, we found palliative care to be provided to only 2 out of 76 severely diseased PD patients (2.6%). 72% of the patients expressed an unmet need for information concerning palliative care, especially about advance care planning concerning end-of-life care (EoLC). In more than 40% of the patients, there had been no discussion about EoLC in the family. Almost half of the patients preferred to consult with their general physician or outpatient neurologist about palliative care matters (Figure 2). The majority of patients wishes to die at home. However, it remains challenging to simultaneously receive professional palliative and neurological care guaranteeing fair symptom control in order to honor this wish in dignity (Figure 2).

**Table 2:** Palliative care implementation in geriatric advanced Parkinson’s disease patients (*n* = 76).

	Yes (%)	No (%)
Advance directive	69.7	30.3
Health-care proxy	68.4	31.6
Actual palliative care	2.6	97.4
Need of information concerning palliative care	72	28
Discussion about end-of-life care in the family	57.9	42.1

## Discussion

By applying the following inclusion criteria, we defined a palliative care intervention phase of advanced PD according to Saleem et al. (15): H&Y 3 or more, 65 years of age or older, disease duration of at least 5 years, and loss of autonomy due to PD. Patients’ parameters such as treatment plans and equivalence dosage of levodopa were in line with earlier reports of long-term surviving PD cohorts from Australia (24) and the US (13). However, patients recruited in our study were older (75.5 years) compared to the Sidney cohort (71 years) (24) and the US cohort (69.5 years) (13). Our patients presented with a higher mean H&Y and a mean disease duration of 17 years, which was 2 years longer than in the study of Hely et al. from 2005 (24). We specifically aimed to measure a geriatric population and, thus, set the inclusion criteria for age at 65 years or older to recruit a more homogeneous group as PD patients seem to reach the palliative care intervention phase of PD in the age of 65 and older (25).

Cognitive deficits were prominent in all comparable cohorts and were impacting HRQOL, nevertheless the prevalence of cognitive deficits and dementia measured by the MoCA Test was dramatically increased in our group of patients compared to the other cohorts. Hely et al. (24) found 48% of the long time PD patients to be demented, measured by the Mini-Mental State Test, whereas Hassan et al. (13) reported a mean MoCA score of 22.6 in their cohort. In our cohort, the MoCA score was markedly lower with a mean value of 18.4 points. We measured HRQOL with the well-established and validated PDQ-39 form (26). In all comparable cohorts, quality of life is decreased significantly and the reduction is related to the duration of disease (2, 13, 24, 27). Hassan et al. (13) described a decrease of 35.8% in quality of life measured in the PDQ-39, which was less prominent than in our cohort (50.8% and SD 12.4%). Considering the higher mean age, comparably long disease duration and extraordinary high prevalence of cognitive decline, it seems likely for our patients to have markedly decreased HRQOL. In our collective of advanced PD patients, we identified motor symptoms, cognitive decline, depression, hallucinations, anxiety, and impairment in the activities of daily living as main factors significantly correlating with decreased HRQOL. In accordance to other studies motor symptoms, cognitive deficits, depression, and psychiatric symptoms showed the strongest correlation with decreased HRQOL (2, 3, 6, 11, 12, 27). In regard to motor complications of advanced PD patients, the most dominant factor in our study appears to be the amount of off-time per day. We also found off-dystonia to negatively impact HRQOL. Interestingly, dyskinesias did not show a significant correlation with HRQOL in our study. It seems that dyskinesias did not or only mildly impact HRQOL in advanced PD (28–30). Therefore, treatment of motor complications should specially aim to reduce off-time of PD patients in advanced stages.

Former studies in advanced PD did not investigate possible benefits of invasive therapies such as DBS or duodopa pump therapy in a palliative setting, which is why we compared HRQOL in patients receiving DBS or duodopa to patients treated with oral medication only. Despite a previously reported positive effect on quality of life of DBS (31) and duodopa treatment (32) for individual patients, we were not able to show a higher HRQOL in our small DBS and duodopa groups compared to patients receiving oral PD medication only.

By considering comorbidities of advanced PD patients, we found cardiovascular disease and hypertension to be the most frequent systemic diseases in their medical history. Generally, PD patients suffer less often from cardiovascular disease and hypertension (33). PD patients may have a special cardiovascular profile of comorbidities compared to the general population possibly caused by peripheral autonomic disturbances. In the context of palliative care, it is important to notice that the cause of death in PD patients is pneumonia to a huge proportion (34), whereas the frequency of cardiovascular and cerebrovascular causes of death is reduced to values of control populations (33, 35, 36). Therefore, we would expect only a limited effect of cardiovascular disease and hypertension on the HRQOL of our PD cohort.

As shown in our data and by others, HRQOL is dramatically decreased in the palliative intervention phase of PD (13, 24). Palliative care may help to sustain or even improve quality of life in these patients by targeting specific symptoms, especially those determining poor HRQOL (14, 17). Only 2 of our 76 patients received palliative care at all, which is in line with observations of Saleem et al. (15) who also found rare implementation of palliative care in advanced PD patients in the UK. Reason for that might be a lack of awareness of clinical criteria when and how to initiate palliative care in PD (19, 37). In our study, a high number of patients reported the wish to die at home, which is in accordance with previous publications (16, 38). However, neurologists and general physicians may not be fully aware of the increased mortality of PD patients and consequently fail to duly address advance care planning during their consultations (39). It is necessary to encourage patients and their caregivers to discuss EoLC and note life-sustaining treatment orders for better care planning according to their individual wishes (40, 41). In regard to the high prevalence of cognitive decline in our cohort, an early advance care planning is of fundamental importance. Additionally, health care by proxy should be discussed in early course of the disease. In later disease stages, decision-making related to goal of care could be compromised by cognitive impairments (42). For excellent reports of advance directives in patients with dementia in the context of ethical and law issues, see Ref. (43, 44). Clearly, defined clinical criteria indicating a time point for palliative care implementation might help to improve future treatment for advanced PD patients. Richfield et al. have defined possible mile stones for initiation of EoLC such as swallowing problems, recurrent infections, marked decline in physical function, first aspiration pneumonia, cognitive difficulties, weight loss, and significant complex symptoms (37, 45). With occurrence of these symptoms, palliative care interventions are helpful and initiation of EoLC should be evaluated.

**Figure 3:**
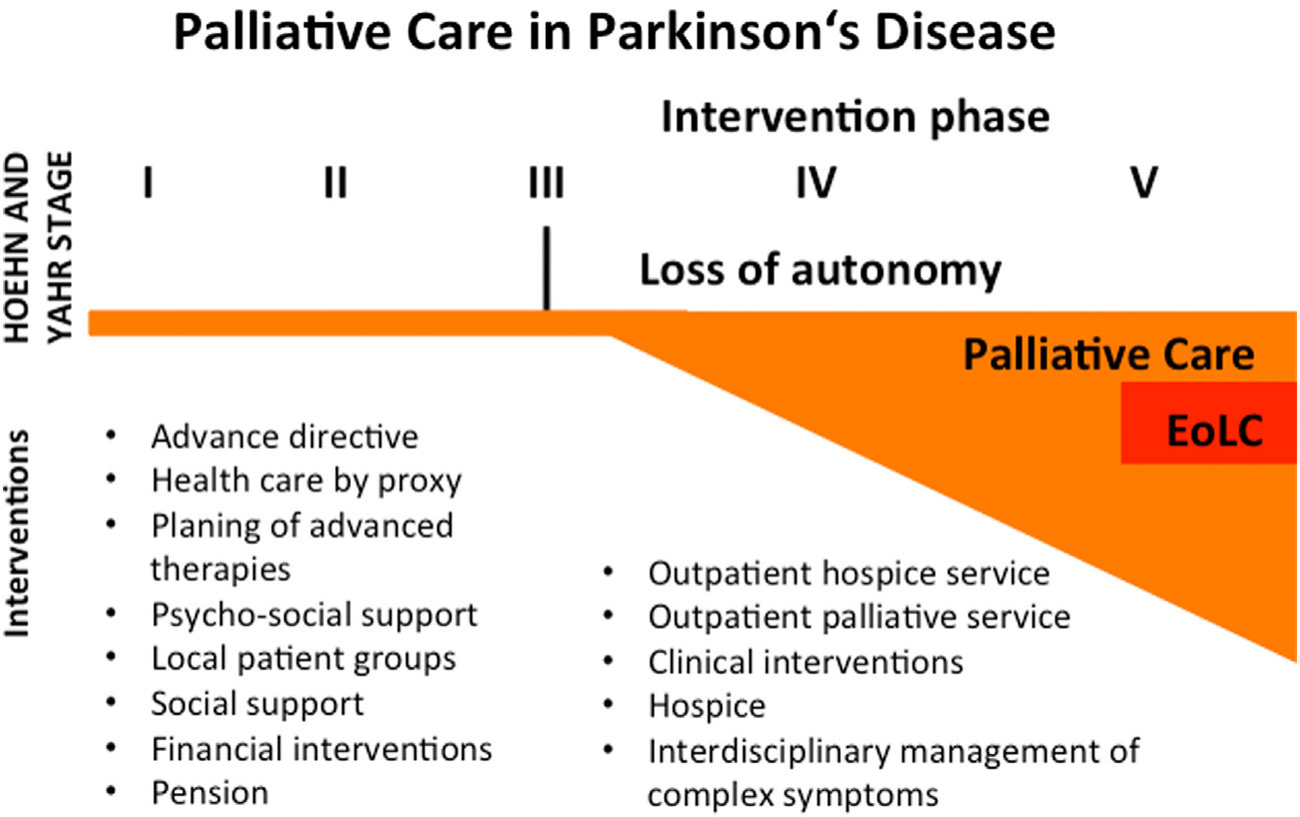
Concept for palliative care in Parkinson’s disease (PD). PD is not curable these days, so therapy is symptomatic. Despite a limited palliative aspect in the beginning of the disease course, patients should be encouraged to write an advance directive or health care by proxy. Informed consent should be guaranteed concerning potential invasive therapies in the future. Local patient groups can help to stabilize the patient in many ways. Physicians should focus on the need for psycho-social and financial support and matters of pension. If patients reach Hoehn and Yahr stage 3 or more, they often lose their autonomy and become dependent on others help. In this phase, palliative care interventions should be initiated. These interventions may be the implementation of outpatient services, an interdisciplinary management of complex symptoms, clinical interventions (e.g., i.v. antibiotics for infections, feeding tubes, airway management, palliative sedation), and discharge to a hospice. Even in the palliative intervention phase, the end-of-life care (EoLC) represents only a small proportion of palliative therapies.

Especially in Germany, we are just at the beginning of properly providing palliative care for neurological patients in general. To serve that purpose, it seems constructive to form interdisciplinary teams (e.g., neurologist, palliative care specialist, PD nurse, and social worker) following the model of Miyasaki et al. in Canada (14). Thereby, the awareness of neurologists concerning palliative care in advanced PD could be improved. Considering our data and recent literature (15, 16), we suggest the following model for palliative care in PD (Figure 3).

Possible limitations of our study are the monocentric approach with a moderately high number of patients (*n* = 76) as well as the restrictive inclusion criteria defining a severely burdened subgroup of PD patients. Due to the evaluation of numerous patient characteristics and extensive neurological examination, it did not seem feasible to apply additional and more specific questionnaires for depression and anxiety. However, for a general and fast assessment of anxiety and depression, the items of the MDS-UPDRS part I can be used in clinical practice (46). We plan the detailed investigation of this issue in a future study explicitly focusing on depression and anxiety symptoms based on our recent results.

In our study, we found that palliative care is not yet a fixed component of PD treatment which is in line with general observations in German PD patients, although implementation of palliative care in advanced PD can be crucial and is often called for. This characterization of severely diseased PD patients contributes novel clinical data and forms the basis for further trials aiming to improve palliative care implementation in advanced PD patients in order to establish optimal symptom control, sustain quality of life, reduce caregiver burden, and prevent caregiver burnout (10). Our data emphasize the urgent need of palliative care in geriatric advanced PD patients.

## Ethics Statement

We obtained approval from the local Ethics Committee of Hannover Medical School (No. 3123-2016), and patients or their caregivers gave written informed consent.

## Author Contributions

MK and FW planed the study. AT, MK, LM, LP, and DD recruited the patients. MK, AT, LP, and FW analyzed the data. MK, AT, LM, LP, CS, DD, and FW wrote and corrected the manuscript.

## Conflict of Interest Statement

The authors declare that the research was conducted in the absence of any commercial or financial relationships that could be construed as a potential conflict of interest. The reviewer SP and handling Editor declared their shared affiliation.
